# Research on Bonding and Shrinkage Properties of SHCC-Repaired Concrete Beams

**DOI:** 10.3390/ma13071757

**Published:** 2020-04-09

**Authors:** Penggang Wang, Maopeng Jiao, Chunhong Hu, Li Tian, Tiejun Zhao, Dongyi Lei, Hua Fu

**Affiliations:** 1 School of Civil Engineering, Qingdao University of Technology, Qingdao 266033, China; wangpenggang@qut.edu.cn (P.W.); jiaomp1994@163.com (M.J.); tlsxf@163.com (L.T.); ztjgp@qut.edu.cn (T.Z.); dongge0379@126.com (D.L.); fs215379@163.com (H.F.); 2 Cooperative Innovation Center of Engineering Construction and Safety in Shandong Blue Economic Zone, Qingdao 266033, China; 3 School of Civil Engineering, Henan Polytechnic University, Jiaozuo 454000, China

**Keywords:** strain hardening cement-based composite (SHCC), repair, shrinkage, cracking, delamination

## Abstract

Traditional cement-based repair materials are brittle and prone to cracking. The failure of more than half of repaired concrete structure is due to the re-cracking of the repair material itself or delamination and peeling from the concrete matrix. Thus, a second repair is required in a short period, increasing the maintenance cost. To reduce cracking, Strain Hardening Cement-based Composite (SHCC), with strain hardening and multiple cracking property, is prepared to study the influence of interface roughness and repair layer thickness on the shrinkage, cracking and delamination modes of SHCC-repaired concrete beams. The results show that under the shrinkage stress, multiple fine cracks instead of local fractures occur in the SHCC repair layer, and the interfacial delamination is effectively controlled. Interfacial bonding property is the main factor that affects the shrinkage and deformation coordination of SHCC-repaired beams. When the interface roughness is different, the crack width of the SHCC repair layer is similar. However, it has a greater influence on the interfacial delamination length and maximum delamination height of the repaired beam. With the increase of interface roughness, the delamination length and height of the repaired beam are greatly reduced. Therefore, before using SHCC to repair the existing structures or components, the bonding surface should be roughened to improve the bond strength between SHCC and the old concrete. With the increase of the repair layer thickness, the cracking and delamination of the repair layer tend to be alleviated. Although the crack width of the repair layer can be effectively controlled after cracking, the overlarge shrinkage (985.35 × 10^−6^, about twice the shrinkage value of ordinary concrete) of the SHCC prepared in this research results in the cracking of the repair layer and the delamination of the repair interface under the restraint of concrete; thus, SHCC fails to repair the concrete efficiently. In terms of shrinkage deformation control, materials with high toughness and low shrinkage are required to repair the existing concrete structures. The implication of this research may provide a theoretical basis for the preparation and application of SHCC with high toughness and low shrinkage.

## 1. Introduction

In recent years, the cost for infrastructure repair and maintenance in developed countries such as the United States, Britain, Japan, and Germany have been increasing, and investment in repair and maintenance of old buildings has accounted for more than 50% of the total construction. In 2018, the United States released the “Legislative Outline for Rebuilding Infrastructure in America”, hoping to use $200 billion in federal government funds to leverage $1.3 trillion in local government and private capital to repair and renew old infrastructure [1]. Since the 1990s, civil engineering projects have continued to grow rapidly in China. Now, many buildings, bridges, and tunnels have entered the maintenance period. A survey by the Ministry of Construction shows that most industrial buildings require repairs after 25–30 years and the service life of buildings in severe environments such as marine environment is only 15–20 years. According to the results of the corrosion survey conducted by Hou Baorong, academician of the Chinese Academy of Engineering, China’s annual loss due to corrosion accounts for about 3.34% of GDP, totaling more than 2 trillion yuan, and one Chinese person has to bear the corrosion cost of 1550 yuan [2]. It is estimated that in the near future, the repair cost of existing concrete structures in China will exceed that of new buildings.

At the early stage, ordinary cement paste, mortar or ordinary concrete were used as repair material of concrete structures. But cracks or delamination often occurred at the interface between the repair layer and old concrete, causing the interface to de-bond. In order to improve the interface performance between repair layer and old concrete, steel fiber, PAN-based carbon fiber, polypropylene fiber, fly ash, silica fume, latex, expansion agent were added to cement-based repair materials [3,4,5,6]. This improves the interface bonding performance to a certain extent, but the type and proportion of additives have a greater impact on the bonding effect of the interface. When using it, attention should be paid to adjusting the content of each material to find the optimal material content. Besides, the effect of polymers on the interfacial bonding properties of repair layer and old concrete was also studied. Judge et al. [7] added styrene-butadiene latex and vinyl acetate to the repair mortar, and found that the bonding property between repair layer and old concrete was improved. Sasse et al. [8] found that a chemical reaction would occur between the resin and cement, which would cause the resin to soften. Frigionc et al. [9] pointed out that water has a great influence on the interfacial bonding performance between concrete and epoxy resin, especially in the long-term impregnation environment, which can lead to the degradation of mechanical properties. More important, traditional cement-based materials are brittle and prone to cracking. Once cracked, the crack width can reach 0.5–3 mm, providing pathways for harmful ions into the materials and thus accelerating the deterioration of structures [10,11,12,13,14]. However, more than half of the repaired concrete structures have failed due to the re-cracking of the repair material itself or the delamination and peeling from the concrete matrix [15,16]. If effective protective measures can be taken, it can reduce the energy and resource consumption caused by demolition or repeated repairs in a short period, as well as negative social impacts such as road congestion and increased pollution. Therefore, it is of great significance to repair existing concrete structures with superior materials and appropriate methods.

SHCC is usually composed of cement, water, fiber, fine aggregate, fly ash, silica fume (microsilica), etc. However, the use of silica fume is often risky in the aspect of its application to SHCC, because this additive of silica fume usually increases the brittleness of the cement matrix [17,18]. SHCC has excellent crack resistance and permeability, owning obvious advantages than other repair materials of concrete structures. The ultimate tensile strain of SHCC can reach 3–7%, which is 300–700 times of ordinary concrete. The crack width of SHCC can be controlled within 100 μm under the ultimate tensile load. In recent years, researchers have carried out a large number of theoretical and experimental studies on the mechanical properties and durability of SHCC [19,20,21,22,23]. The excellent tensile properties and ultra-high toughness of SHCC significantly change the brittleness of traditional cement-based materials, and its good crack control ability is very conducive to the requirement of crack width control in the repair of concrete structures [17]. In the repaired concrete structures, the shrinkage of the existing concrete has been basically completed, while the newly poured SHCC repair layer would shrink during the hardening process, and the volume change caused by the shrinkage would inevitably affect the deformation coordination of the repaired concrete structures. Therefore, it is particularly important to study the shrinkage and deformation coordination of SHCC in repairing existing concrete structures. In this paper, SHCC with strain hardening and multiple cracking property was first prepared. Then the free shrinkage of SHCC and concrete was studied. Finally, the influence of interface roughness and repair layer thickness of SHCC on restrained shrinkage, cracking, and delamination modes of SHCC-repaired concrete beams was studied. Results may provide a theoretical basis for using SHCC to repair existing concrete structures.

## 2. Experimental Program

### 2.1. Experimental Material

The repaired beam is composed of old concrete and repair materials. The mix proportion of concrete with compressive strength of is shown in Table 1. Ordinary Portland cement P O 42.5, river sand with a maximum particle size of 5 mm, 5–20 mm continuous graded aggregate, and tap water were used.

SHCC with compressive strength (100 mm × 100 mm × 100 mm) of 48.49 MPa at the age of 28 days curing in an indoor environment (T = 25 ± 3 °C, RH = 60 ± 5%) was used as the repair material. The mix proportion of SHCC, which is the result of optimization with local materials, is shown in Table 2. Ordinary Portland cement P.O 42.5 and a local fly ash with chemical compositions (determined by X-ray Fluorescence) shown in Table 3 were used. Sand with a maximum grain size of 0.3 mm was used. At the same time, PVA fibers with properties (provided by the manufacturer) shown in Table 4 were added to the fresh mix. In order to improve workability of the fresh SHCC, a small amount of super plasticizer was added. 

### 2.2. Uniaxial Tensile Test of SHCC

**Figure 1:**
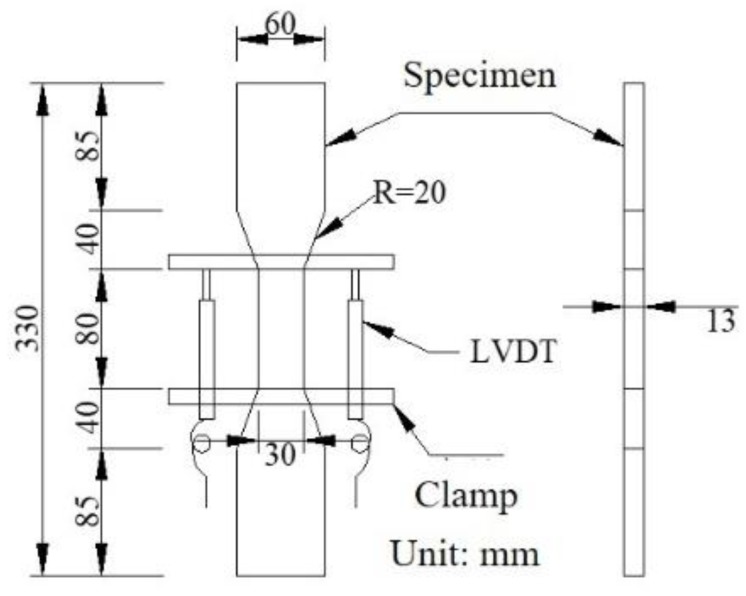
Dumbbell specimen.

The specimens for uniaxial tensile tests of SHCC are dumbbell specimens, as shown in Figure 1. The fresh mix was cast into steel forms. After hardening for 24 h under wet burlap, the forms were removed and the specimens were placed in an indoor environment for curing (T = 25 ± 3 °C, RH = 60 ± 5%). At an age of 1 d, 14 d, 28 d, and 90 d, the uniaxial tensile test was performed under displacement control with 0.005 mm/s on a universal testing machine. 

### 2.3. Free Shrinkage Test

**Figure 2:**
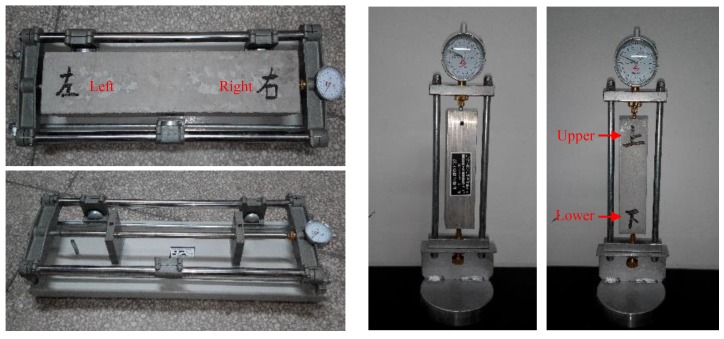
Free shrinkage measurement of SHCC and concrete.

The dimensions of SHCC specimen and concrete specimen for free shrinkage tests are 40 mm × 40 mm × 160 mm and 100 mm × 100 mm × 515 mm. After hardening for 24 h under wet burlap, the forms were removed and the specimens were placed in an indoor environment (T = 25 ± 3°C, RH = 60 ± 5%) for the shrinkage test as shown in Figure 2.

### 2.4. Interfacial Bonding and Shrinkage of SHCC-Repaired Concrete Beams

**Figure 3:**
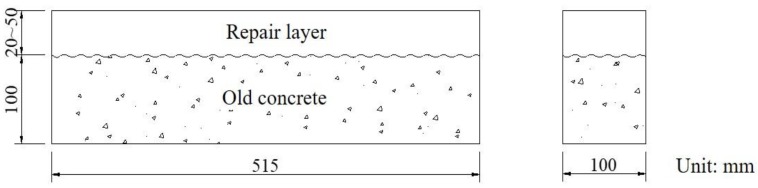
Schematic diagram of SHCC-repaired concrete beam.

The dimension of old concrete beam is 515 mm (length) ×100 mm (width) × 100 mm (height). The dimension of SHCC repair layer is 515 mm (length) × 100 mm (width) with a thickness of 20–50 mm, as shown in Figure 3.

This test focuses on the influence of interface roughness and repair layer thickness on the performance of SHCC-repaired concrete beam as shown in Table 5.

**Figure 4:**
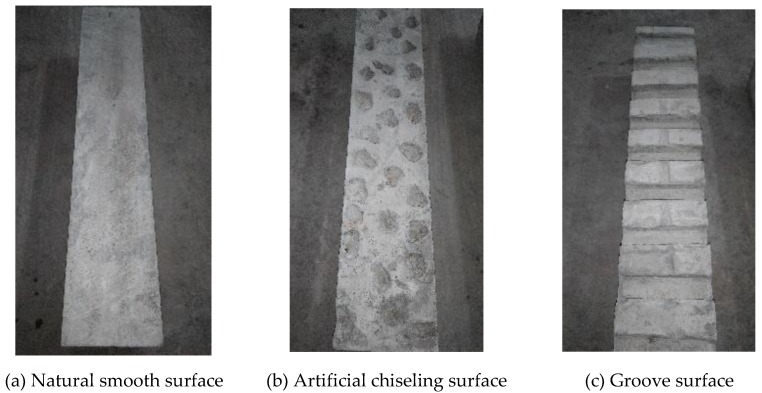
Bonding surface of old concrete after treatment.

**Figure 5:**
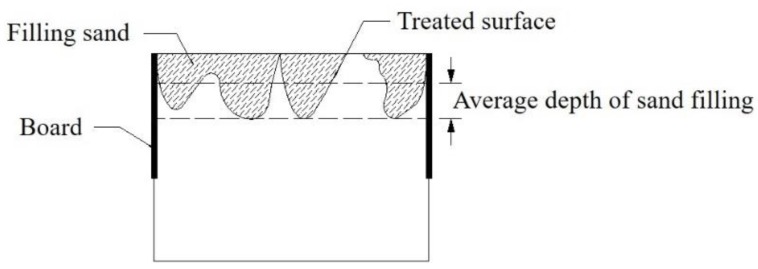
Schematic diagram of the sand filling method.

The casting process of SHCC-repaired concrete beam is as follows:

(1) Preparation of old concrete beams: the old concrete beams were casted first, and the mold was removed after 24 h. Then, the old concrete beams were covered with a plastic film and regularly sprinkled for curing. After 28 days of sprinkling curing, they were kept indoors (T = 25 ± 3 °C, RH = 60 ± 5%) for natural curing until 6 months. The compressive strength of the cubic concrete specimen (100 mm × 100 mm × 100 mm) curing in the same environment for 6 months is 41.04 MPa.

(2) Treatment of the old concrete bonding surface: the old concrete bonding surface during casting was treated as shown in Figure 4. (1) Natural smooth surface: after the old concrete was poured, use a spatula to smooth the upper surface. This type of surface is relatively smooth, as shown in Figure 4a. (2) Artificial chiseling surface: a certain number of stones with a diameter of about 20 mm were randomly pressed into the old concrete. The indentation depth was about half of the stone diameter, and it was then pulled out after 18 h (simulating artificial chiseling), as shown in Figure 4b. This treatment can effectively reduce the disturbance of artificial chiseling on the old concrete surface and prevent microcracks and local damage. (3) Groove surface: the groove method was used to treat the old concrete surface (simulating the cutting groove method). The depth of each groove was about 1/4 to 1/2 of the largest aggregate particle size of the old concrete. The average groove width was 1 to 1.5 times of the largest coarse aggregate particle size of the old concrete [24]. Therefore, the groove size was 100 mm (length) × 20 mm (width) × 10 mm (depth), and the distance between grooves was 35 mm. Before the old concrete was poured and formed, the prepared wooden blocks were pressed into the corresponding positions on the concrete surface and then pulled out after 18 h, as shown in Figure 4c.

(3) The sand filling method was used to evaluate the roughness of the old concrete bonding surface [24], as shown in Figure 5. The average sand filling depth can be calculated according to Equation (1). And the average depth of sand filling of each repaired concrete beam is shown in Table 5.

(1) h=V/A

Where *h* is the average depth of sand filling (mm); *V* is the sand volume (mm^3^); *A* is the area of the cross-section (mm^2^).

**Figure 6:**
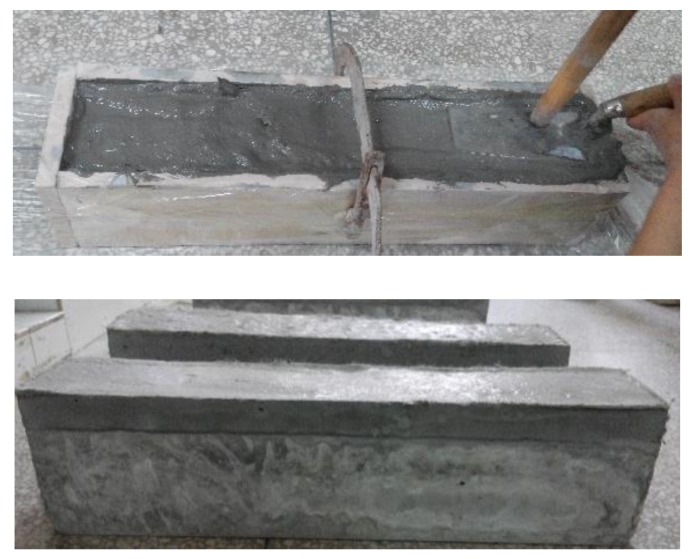
Repaired concrete beam after pouring SHCC layer.

(4) The repair layer was poured following the design requirements in Table 5. Before pouring SHCC, brush the floating ash on the old concrete bonding surface with a wire brush and rinse it with water. Then, sprinkle water on the surface and cover it with a wet towel for 10–12 h to ensure that this surface was saturated. To prevent the wood formwork from absorbing water of the newly poured SHCC, the surface of the wood formwork was coated with a plastic film before SHCC was poured. The repaired beam after pouring SHCC and removing the mold is shown in Figure 6.

(5) Then, the time of the first crack, the crack width (determined by crack width measuring instrument), the length of the cracks (determined by ruler), the number of the cracks were recorded. At the same time, interfacial delamination of SHCC-repaired beams, including delamination position, delamination time, delamination length and delamination height was also recorded.

## 3. Results and Discussion

### 3.1. Uniaxial Tensile Properties of SHCC

#### 3.1.1. Stress–Strain Curves of SHCC under Uniaxial Tension

**Figure 7:**
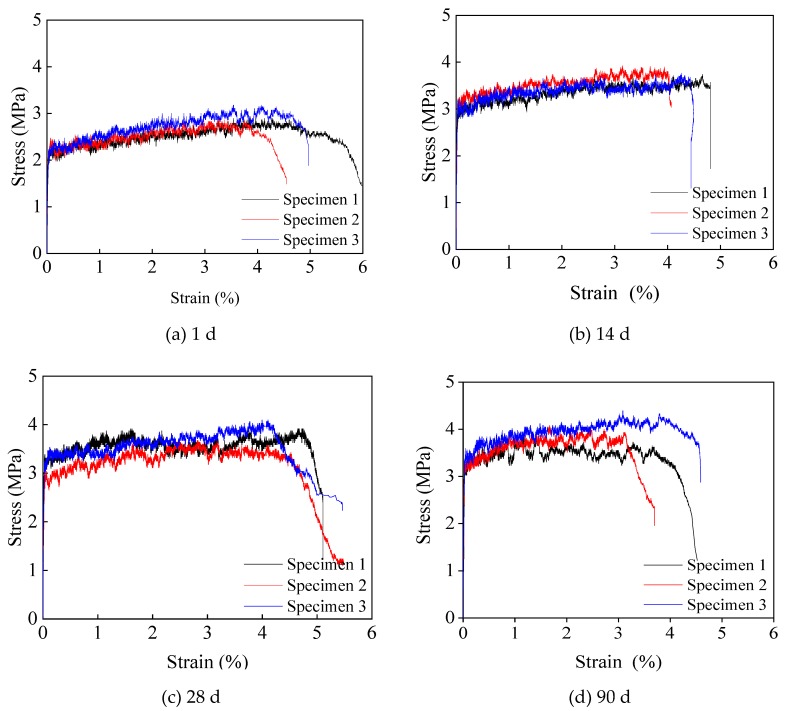
Stress–strain curves of SHCC at different curing ages

The stress–strain curves of SHCC under uniaxial tension are shown in Figure 7. It can be seen that the prepared SHCC specimens at all curing ages show significant strain hardening characteristics, and their ultimate tensile strains all exceed 3%. The stress–strain curve can be divided into three sections: elastic, strain hardening and strain softening sections. (1) The elastic section is from the beginning of loading to the point when the first crack occurs. The stress is proportional to the strain, the slope of which represents the elastic modulus of the specimen. During this stage, the matrix is subjected to external loads. (2) The strain hardening section is when the tensile stress reaches the first cracking strength and the first crack appears in the SHCC specimen. At this time, the PVA fiber there spans between the cracks, and its bridging stress is greater than the cracking strength of the matrix. The load is continuously transmitted to the matrix near the cracks, and the stress is redistributed into the steady-state cracking stage. With the increase of tensile strain, the stress increases slightly, showing significant strain hardening characteristics. Also, the stress–strain curve at this stage fluctuates up and down according to a certain law. Each of these fluctuations represents the appearance of new cracks. (3) In the strain softening section, the crack in the weakest part of the specimen begins to open and form the main crack. The crack width increases significantly, and the fibers are constantly pulled out or broken. With the increase of strain, the stress gradually decreases, showing the strain softening phenomenon. Crack further propagates, which eventually causes the specimen to break. 

From Figure 7, we can obtain the first cracking strength *F_t_*, first cracking strain *ε_t_*, elastic modulus *E_t_*, ultimate tensile strength *f_tu_* and ultimate tensile strain *ε_tu_* of SHCC under tensile load as shown in Table 6. The first cracking strength *F_t_* is the stress value corresponding to the endpoint of the elastic stage of the tensile stress–strain curve. The first cracking strain *ε_t_* is the strain value corresponding to the initial cracking strength. The tensile modulus *E_t_* is the slope of the elastic stage of the stress–strain curve. The ultimate tensile strength *f_tu_* is the stress value corresponding to the highest point on the stress–strain curve. The ultimate tensile strain *ε_tu_* is the strain value corresponding to the softening point on the stress–strain curve. It can be seen from Table 6 that: (1) Both *F_t_* and *f_tu_* increase with the increase of curing age, with rapid growth in the early stages and slow growth in the later stages. The ultimate tensile strength at different ages is about 1.2~1.4 times the first cracking strength. (2) Before 14 days, *ε_t_* and *ε_tu_* increase with the increase of curing age; while after 14 days, *ε_t_* and *ε_tu_* decrease as the curing age increases. (3) *E_t_* increases with the increase of curing age, with rapid growth in the early stages and gradually stable growth in the later stages. (4) The reason for the above results is as follows: with the hydration of cement, both the strength of matrix and the bond strength between PVA fiber and the matrix increase. At the early curing age, the strength of matrix is much smaller compared with the bond strength. If the tensile stress of SHCC is bigger than the strength of matrix, crack occurs. At the same the PVA fiber here spans between this crack. Because the bond strength between PVA fiber and the matrix is bigger than the tensile stress, the PVA fibers can transfer the load to the matrix near this crack. As a result, the tensile strain is increasing. In addition, with the increasing of curing age, the strength of matrix is almost the same with, but still less than the bond strength between PVA fiber and the matrix. In this situation, some of the PVA fibers cannot transfer the load to the matrix near the crack, resulting in the decrease of strain capacity.

#### 3.1.2. Crack Pattern of SHCC at Different Ages

**Figure 8:**
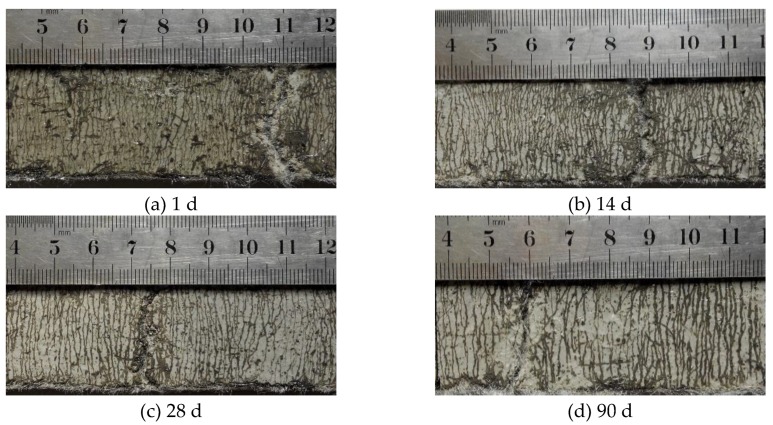
Crack patterns of SHCC specimens under uniaxial tension at different ages.

Crack patterns of SHCC under uniaxial tension at different ages are shown in Figure 8. Obviously, all specimens crack stably under tension. Before the failure, the whole specimen is covered with evenly distributed cracks and shows significant multi-micro cracking characteristics, indicating that the prepared SHCC specimen has an excellent capacity of crack control. However, as the curing age increases, the crack width of SHCC specimen increases (the average crack width increases from 40 μm to 48 μm), the number of cracks decreases, and the crack spacing gradually increases (the average crack spacing increases from 0.941 mm to 1.348 mm).

### 3.2. Free Shrinkage of SHCC and Concrete

**Figure 9:**
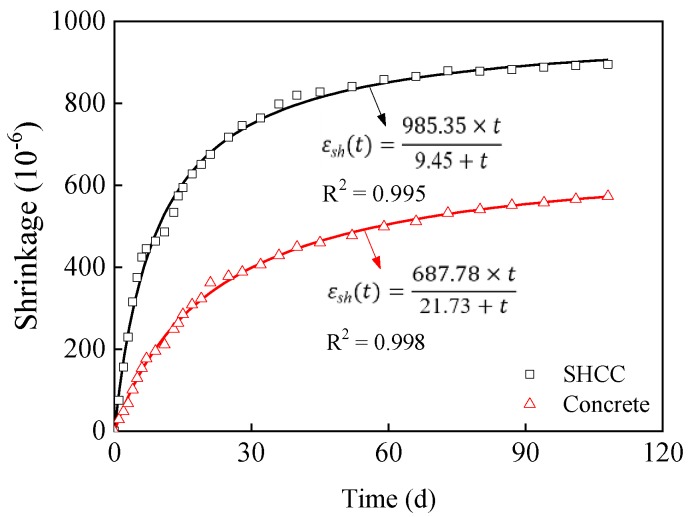
Drying shrinkage of SHCC and concrete.

Figure 9 shows free shrinkage of SHCC and concrete in an indoor environment (T = 25 ± 3 °C, RH = 60 ± 5%). Obviously, the shrinkage value of the specimen increases with drying time. This is because, during the indoor curing process of the specimen, cement furtherly hydrates, consuming the internal water and thus reducing the moisture content of the specimens. Moreover, the relative humidity inside the specimen is greater than that of the indoor environment, and there is a moisture gradient inside and outside the specimen. This lead to moisture evaporate from the specimen to the indoor environment. Under the combined effects of cement hydration water consumption and water evaporation, the relative humidity inside the specimen decreases, resulting in an increase in the surface tension of the capillary pore solution of the specimen. At the same time, the disjoining pressure between C-S-H gel decreases, the van der Waals force increases, and the specimen shrinks. However, the shrinkage value of SHCC is much larger than that of concrete at the same drying time. This is because in the design of SHCC, in order to obtain strain hardening and multi-micro cracking characteristics, the amount of sand needs to be reduced, and coarse aggregates cannot be used, resulting in the increased shrinkage of SHCC. Researchers have taken various measures to reduce the drying shrinkage of SHCC. Li et al. [25] found that low alkali cement can effectively reduce the drying shrinkage of SHCC. Zhang et al. [26] have developed SHCC with high toughness and low shrinkage by improving the SHCC matrix. The drying shrinkage of the prepared SHCC by Zhang et al. was only 10% ~ 20% of that of traditional SHCC. Sahmaran et al. [27] have found that the reduction degree of the drying shrinkage value of SHCC increases with the increase of light aggregate replacement rate. It can be seen that researchers have studied the impact of various measures on the shrinkage of SHCC from different perspectives, but the research is not systematic, indicating the necessity of conducting in-depth systematic research.

Studies show that the shrinkage curve of cement-based materials can be fitted with Hyperbola function, exponential function and logarithmic function [28,29,30,31]. In this paper, Hyperbola function is selected to perform the regression analysis in Figure 9, as shown in Equation (2) [29]. The fitting formula is shown in Figure 9. The fitting correlation coefficients of SHCC and concrete are more than 0.99. It can be seen that the shrinkage curve of SHCC and concrete can be described and predicted by Hyperbola function, and the fitting parameters have clear physical meanings. It can be seen from the fitting results that the final shrinkage values of SHCC and concrete are 985.35 × 10^−6^ and 687.78 × 10^−6^, and the drying time when they reach half of the final shrinkage value are 9.45 days and 21.73 days, respectively, which agrees with the test results.

(2) $$\varepsilon_{t}=\frac{at}{b+t}$$

where *t* is the drying time, day; *a* is the final shrinkage value of the specimen; *b* is the drying time when the shrinkage of the specimen reaches half of the final shrinkage value, day.

### 3.3. Interfacial Bonding and Shrinkage Properties of SHCC-Repaired Concrete Beams

#### 3.3.1. Restrained Shrinkage Stress and Failure Mode Analysis of SHCC-Repaired Concrete Beams

**Figure 10:**
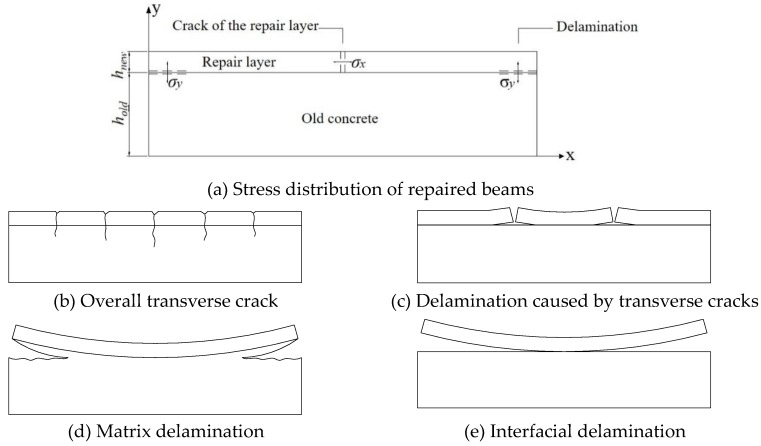
Restrained shrinkage stress and failure mode of SHCC-repaired beams.

The shrinkage of the old concrete in the repaired beam has been basically completed after 6 months curing. The SHCC-repaired layer shrinks after being poured. The deformations of the old concrete and the repaired layer are inconsistent, making the shrinkage deformation of the repaired layer constrained by the old concrete, which generates tensile stress in the repaired layer. Meanwhile, tensile and shear stresses could be generated at the interface between the old concrete and the repair layer. When the tensile stress exceeds the tensile strength of the repair material or interfacial bond strength, the repaired beam is prone to cracking and interfacial delamination. The restrained shrinkage stress and failure mode analysis of the repaired beam are shown in Figure 10 [32]. The tensile stress in the repair layer is x-direction stress. When the tensile stress is larger than tensile strength of SHCC, cracks may occur. If the repair layer is well bonded, the cracks may penetrate to the old concrete, as shown in Figure 10b. Otherwise, the crack will be orthogonal to the interface and delaminate with the matrix, as shown in Figure 10c. The tensile stress at the interface between the repair layer and the old concrete is y-direction stress. When the stress is larger than the bond strength of the interface, the interface may open at the contact surface or inside the old concrete, as shown in Figure 10d. The shear stress at the interface between the repair layer and the old concrete is x-axis stress. When the shear stress is large enough, a poorly bonded component may cause the repair layer to slide along the surface of the old concrete, as shown in Figure 10e.

If the shrinkage deformation of the repair layer does not change along its height, under the restraint of the old concrete, the maximum tensile stress *σ_xx_* in the repair layer would occur in the middle of the repair layer bottom, and the maximum interface tensile stress *σ_xy_* and interface shear stress *σ_yy_* would occur at the end of the repaired beam [33]. In fact, the shrinkage of the repair layer varies in height [34]. Due to the effect of the moisture gradient, the maximum tensile stress appears on the surface of the repair layer. As the shrinkage progresses, the tensile stress increases. When the maximum tensile stress exceeds the tensile strength (or the maximum tensile strain exceeds the ultimate tensile strain) of the repair layer, cracks appear on the surface of the repair layer. As the shrinkage progresses, the crack would expand downward along the cross section, and the width of the crack on the upper surface would further increase. The moisture gradient could also cause warping at both ends of the repair layer, and tensile and shear stresses at the interface between the repair layer and the old concrete, resulting in the delamination of the repair beam. It can be seen that the middle part of the repair layer is prone to cracking, and the end of the repair beam is prone to delamination. When the bond strength between the repair layer and the old concrete is high, the delamination phenomenon is not apparent, but the repair layer is more prone to cracking, and the stress inside the repair layer and at the interface is redistributed. If the internal stress of the repair layer after cracking is still greater than its tensile strength, cracks would continue to appear in the repair layer, and the crack development would be stable until the stress in the repair layer is less than its tensile strength. When the tensile strength of the repair material is high, the cracking of the repair layer may be delayed, but the risk of interfacial delamination is increased. 

**Figure 11:**
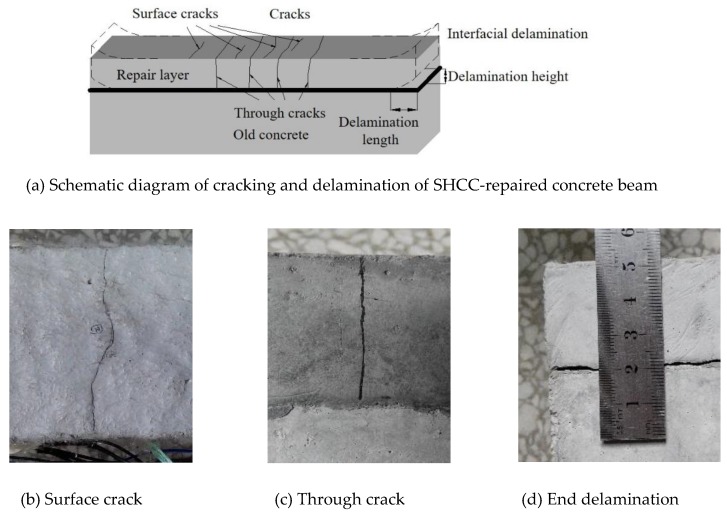
Typical crack formation and interfacial delamination patterns of SHCC-repaired concrete beam.

It is found that under the restraint of the old concrete, SHCC-repaired concrete beams show cracking or delamination or a combination of the two to varying degrees, as shown in Figure 11. Table 7 shows the time of the first crack, the number of cracks, crack width, end interface delamination height and delamination length, and the cracking and delamination time of the SHCC repair layer.

#### 3.3.2. Influence of Interface Roughness on Cracking and Delamination Modes of SHCC-Repaired Concrete Beams

**Figure 12:**
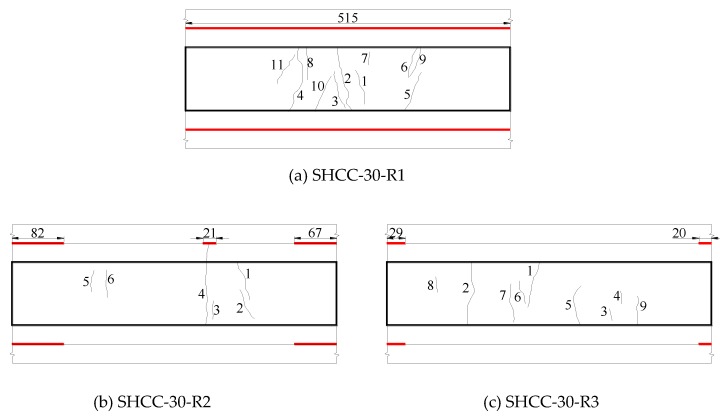
Influence of interface roughness on cracking and interfacial delamination patterns of SHCC repaired beams (mm).

The influence of interface roughness on cracking and delamination modes of SHCC-repaired concrete beam is shown in Figure 12. From Figure 12 and Table 7, it can be seen that the artificially chiseled repaired beam (SHCC-30-R2) has a small number of cracks. In addition to surface cracks, there is a crack that penetrates one side. The maximum crack width is 0.05 mm, and the average crack width is less than 0.04 mm. The repaired beam with a natural smooth surface (SHCC-30-R1) and the grooved repaired beam (SHCC-30-R3) only have surface cracks. Besides, there are many cracks; the maximum crack width is 0.04 mm, and the average crack width is less than 0.04 mm. It can be seen that when the interface roughness is different, the shrinkage crack width of SHCC does not change significantly, and the shrinkage crack can be controlled within 0.05 mm, which is advantageous for durability [35]. Moreover, the repaired beam with a naturally smooth surface is delaminated on the 3rd day after demolding, and the whole layer is delaminated on the 4th day. The maximum delamination height is 2.71 mm. The maximum delamination lengths of the artificially chiseled and grooved repaired beams are 82 mm and 29 mm, and the maximum delamination heights are 0.07 mm and 0.08 mm, respectively. As the roughness increases, the delamination length and height of the SHCC-repaired beams are controlled. The delamination length of the artificially chiseled repaired beam is longer than that of the grooved repaired beam, but its delamination height is smaller.

For SHCC-repaired beams with different interface roughness, compared with the crack damage degree (crack width ≤ 0.05 mm), the delamination damage degree (delamination height ≥ 0.07 mm) is more serious. The effect of roughness on the delamination of SHCC-repaired beams is more significant, and it has less influence on the surface cracking. Under the action of shrinkage tensile stress, the SHCC repair layer is in the stage of tensile strain hardening, and the crack width is controlled due to the bridging of the fiber without local fracture. The SHCC-repaired beam with a naturally smooth surface has serious delamination, indicating that the mechanical bond and friction resistance of the smoother old concrete bonding surface are small. The SHCC repair layer has a larger early shrinkage after pouring, and smaller bond strength between the repair layer and the old concrete leads to delamination.

#### 3.3.3. Influence of Repair Layer Thickness on Cracking and Delamination Modes of SHCC-Repaired Concrete Beams

**Figure 13:**
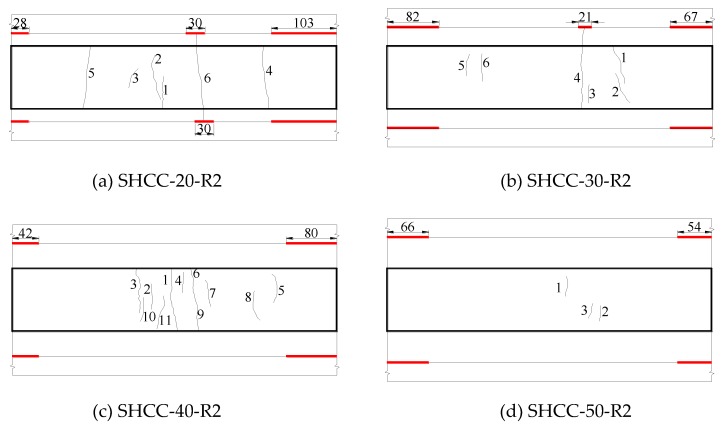
Influence of repair layer thickness on cracking and interfacial delamination patterns of the repaired beams (mm).

The influence of repair layer thickness on the cracking and delamination mode of SHCC-repaired concrete beam is shown in Figure 13. From Figure 13 and Table 7, it can be seen that for the thinner repaired beam (SHCC-20-R2), the shrinkage cracks appear earlier, about on the 7th day. In addition to surface cracks, a through crack also appears with the maximum crack width of 0.05 mm. With the increase of repair layer thickness, the occurrence time of cracks delays 2~6 days, and the number of cracks shows a downward trend. The 30 mm-thick repaired beam (SHCC-30-R2) has a crack through one side, and the rest cracks are surface cracks with a maximum crack width of 0.05 mm. The SHCC-40-R2 and SHCC-50-R2 repaired beams have only surface cracks with a maximum crack width of 0.04 mm. As the repair layer thickness increases, the maximum crack width gradually decreases from 0.05 mm to 0.04 mm, and the average crack width is less than 0.04 mm. When the repair layer thickness is different, under the action of shrinkage stress, SHCC behaves as a multi-micro cracking mode instead of a local fracture failure. Moreover, the delamination of the thinner repaired beam occurs earlier. The delamination of SHCC-20-R2 repaired concrete beam occurs about on the 19th day. With the increase of thickness, the delamination time delays 6 days. The maximum delamination length of 20–50 mm-thick repaired beam is 103 mm, 82 mm, 80 mm, 66 mm, and the maximum delamination height is 0.08 mm, 0.07 mm, 0.08 mm, and 0.07 mm, respectively. Therefore, as the repair layer thickness increases, the delamination length and height tend to decrease.

SHCC-repaired concrete beams of different thicknesses have a slightly increased degree of delamination damage (delamination height of 0.05~0.08 mm) compared with the crack degree damage (crack width ≤ 0.05 mm). When the repair layer has a relatively thin thickness, the moisture transmission pathway between the interior and the outside of the repair layer is shorter, the moisture exchange rate is faster, and the water loss degree is larger, causing the SHCC to shrink, and the repaired concrete beam is more prone to cracking and delamination. When the repair layer thickness (within 50 mm) increases gradually, the degree of cracking and delamination of the repair layer tends to decrease.

## 4. Conclusions

(1) When SHCC is used as the repair material, under the action of shrinkage stress, there are many fine cracks instead of local fractures in the repair layer, and the interfacial delamination is effectively controlled.

(2) The interfacial bonding property is the main factor that affects the shrinkage and deformation coordination of SHCC-repaired concrete beams. When the interface roughness is different, the maximum shrinkage crack width of the SHCC repair layer is 0.05 mm, and the average crack width is less than 0.04 mm. The crack widths are not much different. However, the interface roughness has a greater influence on the interfacial delamination length and maximum delamination height of the repaired concrete beam. With the increase of interface roughness, the delamination length and height of the repaired concrete beam are greatly reduced. Therefore, before using SHCC to repair the existing concrete structures, the bonding surface should be roughened to improve the bond strength between SHCC and the old concrete. However, besides the interface roughness, the interfacial bonding and shrinkage properties of repaired beams are also affected by the properties of the old concrete and SHCC, requiring an in-depth study.

(3) When the SHCC repair layer is relatively thin, the cracking and delamination degree is greater. As the repair layer thickness increases, the cracking and delamination degree of the repair layer tends to decrease. When it is greater than or equal to 30 mm, the downward trend is insignificant. In view of the overall performance and economic benefits of the repaired concrete beams, without affecting the bearing capacity and durability of the repaired beam, a thinner SHCC repair layer is preferred to repair and strengthen the existing concrete structure or component.

(4) The final shrinkage value of SHCC prepared in this paper is about 985.35 × 10^-6^. Although the crack width of the repair layer can be effectively controlled after cracking, the overlarge shrinkage of the SHCC will result in the cracking of the repair layer and the delamination of the repair interface under the restraint of concrete; thus, SHCC fails to efficiently repair the concrete. In terms of shrinkage deformation control, materials with high toughness and low shrinkage are required to repair the existing concrete structures. By combining the advantages of different shrinkage-reducing measures, multiple regulation methods can be adopted to solve the coordinated development of strength, toughness, durability, shrinkage and deformation of SHCC. 

(5) Further studies about the bond property between SHCC and concrete, the mechanical property of SHCC-repaired concrete beams are needed for the design and application of SHCC to repair reinforced concrete structures in sever environment, for example marine environment.

## Figures and Tables

**Table 1:** Mix proportion of the old concrete (kg/m3).

No.	Cement	Sand	Aggregate	Water
Concrete	317	757	1136	190

**Table 2:** Mix proportion of SHCC (kg/m3).

No.	Cement	Fly Ash	Sand	Water	SP	PVA Fiber
SHCC	555	680	490	420	27.8	26

**Table 3:** Chemical composition of cement and fly ash (%).

Material	CaO	SiO_2_	Al_2_O_3_	MgO	SO_3_	Fe_2_O_3_	K_2_O	TiO_2_	MnO	Na_2_O	P_2_O_5_
Cement	57.27	20.60	7.17	4.70	4.43	3.85	0.77	0.40	0.35	0.17	0.13
Fly ash	1.83	58.10	31.79	-	0.51	3.76	1.51	1.57	0.02	0.36	0.20

**Table 4:** Property of PVA fiber.

Length (mm)	Diameter (μm)	Young’s Modulus (GPa)	Elongation (%)	Tensile Strength (MPa)	Density, (g/cm^3^)
8	39	42	7	1600	1.3

**Table 5:** Number of SHCC-repaired concrete beams.

Influencing Factors	No.	Interface Type/Average Depth of Sand Filling (mm)	Repair Layer Thickness (mm)
Interface roughness	SHCC-30-R1	Natural smooth/0	30
	SHCC-30-R2	Artificial chiseling/2.64	30
	SHCC-30-R3	Groove/3.5	30
Repair layer thickness	SHCC-20-R2	Artificial chiseling/2.50	20
	SHCC-30-R2	Artificial chiseling/2.64	30
	SHCC-40-R2	Artificial chiseling/2.27	40
	SHCC-50-R2	Artificial chiseling/2.59	50

**Table 6:** Mechanical parameters of SHCC under uniaxial tension.

Age (day)	*F_t_* (MPa)	*ε_t_* (*%*)	*E_t_* (GPa)	*f_tu_*, (MPa)	*ε_tu_* (*%*)
1	2.050 ± 0.028	0.0237 ± 0.00024	8.645 ± 0.064	2.945 ± 0.143	4.247 ± 0.370
14	2.893 ± 0.125	0.0242 ± 0.00076	11.945 ± 0.489	3.750 ± 0.057	4.336 ± 2.956
28	2.950 ± 0.166	0.0237 ± 0.00055	12.421 ± 0.956	3.824 ± 0.219	4.331 ± 0.306
90	3.073 ± 0.018	0.0223 ± 0.00106	13.795 ± 0.586	3.950 ± 0.291	3.545 ± 0.287

**Table 7:** Crack formation and interfacial delamination of SHCC-repaired concrete beams.

Influencing Factors	No.	Cracks of Repair Layer					Interfacial Delamination			
		Time of the First Crack (d)	Number of Cracks		Crack Width		Delamination Position	Delamination Time (d)	Delamination Length (mm)	Delamination Height (mm)
			Number of Surface Cracks	Number of through Cracks	Average Crack Width (mm)	Maximum Crack Width (mm)				
Interface roughness	SHCC-30-R1	8	11	0	<0.04	0.04	Left Right	3 4	515 515	2.71 0.45
	SHCC-30-R2	9	5	1	<0.04	0.05	Left Right	25 28	82 67	0.07 0.07
	SHCC-30-R3	9	9	0	<0.04	0.04	Left Right	28 28	29 20	0.08 0.07
Repair layer thickness	SHCC-20-R2	7	5	1	<0.04	0.05	Left Right	19 21	28 103	0.05 0.08
	SHCC-30-R2	9	5	1	<0.04	0.05	Left Right	25 28	82 67	0.07 0.07
	SHCC-40-R2	10	10	0	<0.04	0.04	Left Right	25 32	42 80	0.05 0.08
	SHCC-50-R2	13	3	0	<0.04	0.04	Left Right	32 25	66 54	0.05 0.07
